# Production and effects of nanomineral selenium (Nano-Se) feed additive on rumen fermentation, productivity, and reproductive performance of ruminants

**DOI:** 10.5455/javar.2024.k830

**Published:** 2024-09-30

**Authors:** Pradita Iustitia Sitaresmi, Mohammad Firdaus Hudaya, Bayu Andri Atmoko, Wulandari Wulandari, Tri Ujilestari

**Affiliations:** 1 Research Center for Animal Husbandry BRIN, Bogor, Indonesia; 2 Research Center for Food Technology and Processing, BRIN, Yogyakarta, Indonesia

**Keywords:** Nanomineral, ruminant, nano-Se, feed additive

## Abstract

Nanotechnology (Nano) applications of feed additives can potentially improve feed-substrate efficiency to enhance livestock productivity. The utilization of Nano in feed in ruminants still tends to be under-explored and reviewed, particularly the application of Nano in trace minerals to enhance the reproductive performance and productivity of ruminants such as selenium. Trace minerals are essential for animal well-being and productivity, and the bioavailability of trace minerals is influenced by a complex matrix of interacting variables, including the chemical form of the minerals used and those found in the diet, the nature of the food ingested, the total composition of the diet, and the health and nutrition of the livestock. Nanominerals such as selenium (nano-Se) have shown impressive results when used as animal feed supplements in ruminants. Nano-Se can significantly boost wellness and immunity, gastrointestinal system function, microbiota homeostasis, metabolism, and reproductive performance in ruminants. This review aims to present the current knowledge on the technology of nano-Se in ruminants, ranging from the nanomanufacturing procedures of nano-Se, the impact of supplementation on the ruminant digestive system, productivity, and reproductive performance in ruminants in some dosages to find the optimized dosage to be provided.

## Introduction

Nanotechnology (Nano) is a field of science that is being developed in the agriculture sector and has potentially new and exciting technologies to drastically alter the livestock sector [1]. Initially, Nano in the livestock industry was often used only for medical purposes [2–4], but in its development, Nano began to be used as a feed additive to increase livestock performance [5]. The application of Nano in feed additives can potentially improve the efficiency of active substrates and enhance livestock productivity [6]. Nano in feed additives is an approach (different chemical, physical, biological, and hybrid processes) [7,8] to fragment the target feed substrate to nanosize (1–1,000 nm) in a stable formula to expand the substrate surface area and potentially improve physicochemical properties and catalyst function [9–11], then facilitate absorption in the digestive system [12].

The utilization of Nano in ruminant feed remains under-explored [13], particularly the application of Nano in micro-mineral (trace nanomineral) nutrition to enhance the reproductive performance and productivity of ruminants, such as selenium [14,15]. Minerals are vital subtracts in animal productivity and health; ruminant animals most often get their mineral intake from supplementation of additional feed given by farmers because of the natural lack of minerals in the feed [16–18]. Nanominerals such as selenium (Nano-Se) [14,19,20] have shown impressive results when used as a fodder supplement in small ruminants. However, the number of available review articles was still limited. Nanominerals significantly enhance ruminant health, immunity, gastrointestinal function, microbiota balance, metabolism, and reproductive performance compared to natural forms [21–23]. In addition, nanomaterials increase the possibility of increasing the production of functional and safe animal products, potentially reducing the use of antibiotics and increasing the concentration of minerals (in a more natural form of the substrate) in animal products (meat and dairy), which is necessary to improve human well-being [11,24–26].

In general, the effect of selenium and nano-Se are increasing body weight [27], increasing livestock productivity such as increasing yield weight or milk yield [14,28–30], increasing feed intake or total digestible nutrient (TDN) [31], increasing assisted reproductive technology ART efficiency [32] such as improving fresh or frozen semen quality, increasing antioxidant function in livestock semen, improving quality of culture media [28,33–36], increased pregnancy rates [14,37,38], elevated red blood cells [39,40], enhancing the production of protein antibody receptors [41], increasing immunity [42–44], anti-microbial [45,46], and others.

**Figure 1.**
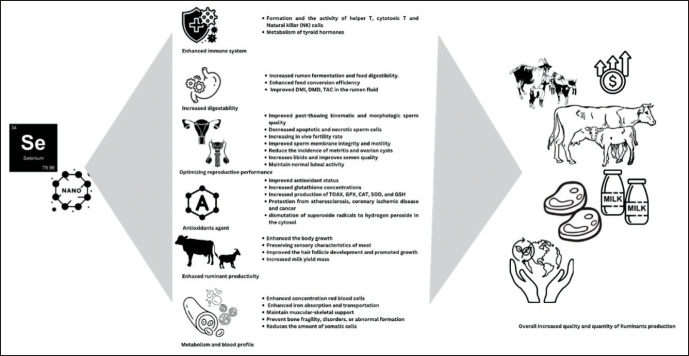
Beneficial and function on nano selenium in ruminants vaccines. The advantages of nano selenium on ruminant performance. Nano selenium has been known to have special functions in maintaining the rumen environment, and increasing livestock productivity, and has the potential to create nano minerals that are more environmentally friendly than their original form.

A brief overview of the functions of nano-Se is presented in Figure 1. Scientists have attempted to optimize the advantages of implementing Nano by incorporating nano-Se into the diet of ruminants and using their benefits to enhance animal performance and health and benefits to ruminants. Therefore, in this review, we have tried to summarize and conclude the beneficial effects of nano-Se on rumen fermentation effects, productivity, and reproductive performance and their possible use as mineral supplements for different categories of ruminants.

## Materials and Methods

### Ethical approval

This manuscript constitutes a review paper; therefore, no ethical clearance is required.

### Methodology of review

This research was conducted using various databases such as Web of Science, PubMed, MEDLINE, and Google Scholar. Over 1,000 scientific publications were reviewed, and the findings from selected research studies (original articles, experimental studies) were compared. Emphasis was placed on original articles, while more than hundreds of review articles were excluded, and 106 articles were suitable for this review. Searches were based on phrases such as selenium nanomineral, nanomineral manufacturing process, Se nanomineral activity in parameters of rumen effects, ruminant productivity, and reproductive enhancement. Searches on recommended doses and toxic thresholds were also reviewed in this paper. This review covers research findings from 2,000 to 2,023.

***Determination and Production of Addition Nano-Se in Ruminants***

Presently, there is substantial interest in the advancement of nanotechnological procedures and approaches that enhance chemical synthesis efficiency. This interest arises from numerous benefits, including the preservation of natural resources, easy manufacturing processes, and long-lasting positive effects on healthcare and ecosystems [47]. In general, minerals like selenium exist in the form of inorganic salts, displaying low absorption. Addressing this issue, one solution is the production of minerals in nanosize. Utilizing nanomaterials with an average size ranging from 1 to 1,000 nm enhances absorption by widening the surface area [48]. The application of nanosized minerals has the potential to enhance the bioavailability of each mineral through increased cellular uptake, mitigating mineral antagonism. This effectiveness depends on the specific fabrication process. Nevertheless, it is crucial to consider the metabolism of these nanominerals within the animal body [1]. Additionally, minerals from inorganic sources have low bioavailability. Supplementing them at levels 20–30 times the animal’s requirements may lead to environmental contamination. Nanominerals enhance bioavailability due to their increased surface area, boosting catalyst efficiency and absorption. Encapsulating these nanominerals aims to prevent interactions among minerals or other nutrients, averting reduced bioavailability [49]. Nanoparticles (Nano) enhance mineral utilization, conserve raw materials, and decrease excreta [50]. However, research on nanomineral fabrication in animals is presently limited.

Nanominerals can be synthesized by physical, chemical, and biological processes [48]. Physical methods include mechanical methods, gas-phase synthesized nanomaterials, and physical vapor deposition. Chemical methods include the effect of reducing agents in the synthesis of Nano, green chemical synthesis, microemulsion methods, stability of metal Nano , synthesis of zero-valence iron, and stabilization by solvent molecules or ligands. Meanwhile, biological methods in nano-synthesis are carried out to reduce toxicity; the method generally carried out is the production with the help of microorganisms or plant-derived materials through intra or extracellular processes, which produce nano-sized biological inorganic materials. Nanominerals can also be synthesized using the top-down method, a physical method by grinding the metal mechanically, or using the bottom-up method, which depends on the wet chemical reduction of mineral salts [51].

### Physical synthesis of nano-Se

Nano-Se are synthesized using physical methods primarily synthesized using ball milling, laser ablation, and pulsed wire discharge. The principle of making nanominerals with this method is to physically break mineral compounds into nano sizes either by friction, impact, or the use of lasers in breaking large compounds into Nano sizes.

### Mechanical procedure or ball milling

**Figure 2.**
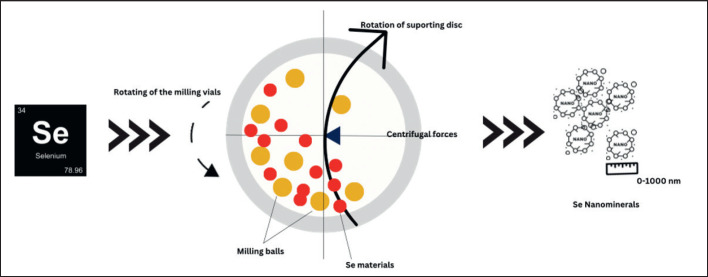
Ball milling nano-Se procedures (adapted by Rane [54]). The manufacture of nano selenium is carried out by putting dry selenium into a ball mill machine that moves at high speed so that the mineral will be physically hit and the resulting mineral is in nanosize.

The milling procedure modifies minerals on the nanoscale in multiple ways and has become the easiest method to produce nano-Se. In this nanomineral synthesis procedure, various milling tools are among the instruments used. Several variables, including milling type, speed, temperature, time, atmosphere, and container, determine the method [52,53]. The chamber is filled with inert gas, physical ball, or air and turned at a high velocity around a centerline axis. Then the mineral is pressed towards the cylinder and against the cylinder wall. The rotation and grinding duration speeds were precisely controlled based on the size of the Nano to be synthesized [6]. Ball milling nano-Se procedures are presented in Figure 2.

**Figure 3.**
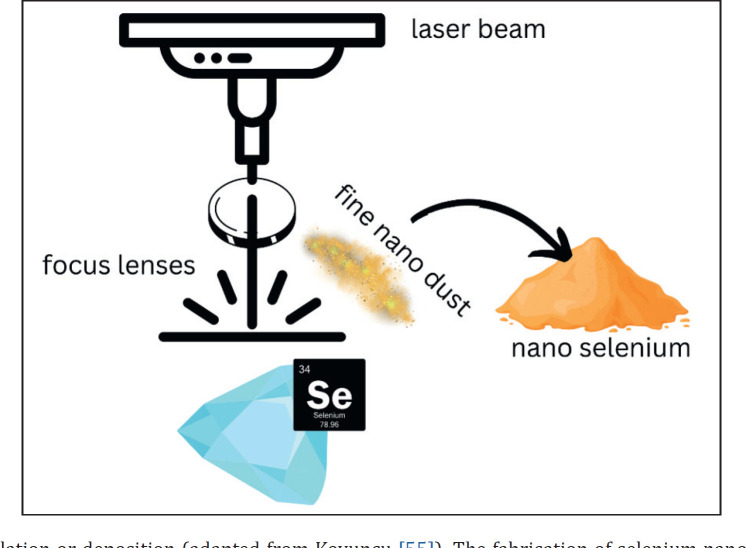
Pulse laser ablation or deposition (adapted from Koyuncu [55]). The fabrication of selenium nano using a laser occurs by placing the selenium mineral under the focusing lens so that the laser will be focused on one firing spot and the nano-fine grains will scatter towards the outside of the target mineral.

### Pulse laser ablation or deposition

Fundamentally laser ablation is devised to produce colloidal Nano in various solvents. Nano-Se are prepared in a hydrated form to avoid oxidation [56]. The laser pulse ablation technique was conducted inside an inert gas vacuum-sealed chamber. In this technique, the final product is influenced by factors such as laser type, pulse duration, and solvent type. Pulse laser ablation or deposition is presented in Figure 3.

### Chemical synthesis of nano-Se

**Figure 4.**
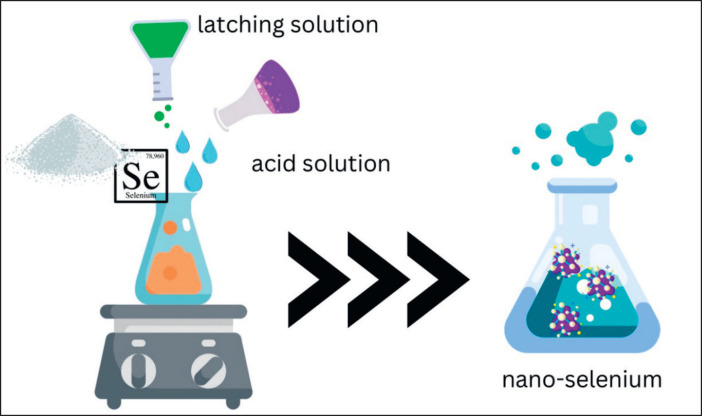
The chemicals method produced nanomaterials (adapted from Aguilar [63]). The principle of making nano selenium chemically is basically by utilizing a low pH solution at a high temperature to rupture mineral bonds so that nanosize is formed, in addition, anti-augmentation substain is also added so that nano selenium granules are expected not to reassemble.

The manufacture of nano-Se can be performed by chemical methods, which provide chemical compounds that are either acidic or basic to reduce minerals so that nano-sized minerals are obtained [48]. Nano-Se can be produced through a redox reaction of ascorbic acid with a Nano -sodium selenite precursor dissolved in a stabilizer with a sodium selenite concentration ranging from 0.03 to 0.012 molarity per liter. Ascorbic acid was then added to sodium selenite at a concentration of 1:1 molar-chitosan (3% weight) dissolved in a 2% succinic acid solution. UV-vis spectrophotometry at wavelengths between 250 and 280 nm was used to observe the formation of nano-Se. The solution was diluted 10 times with distilled water and administered every 30 min. Infrared spectroscopy was used to observe the interactions between selenium and chitosan [57]. The chemical method produced nanomaterials is presented in Figure 4.

### Biological synthesis of nano-Se

**Figure 5.**
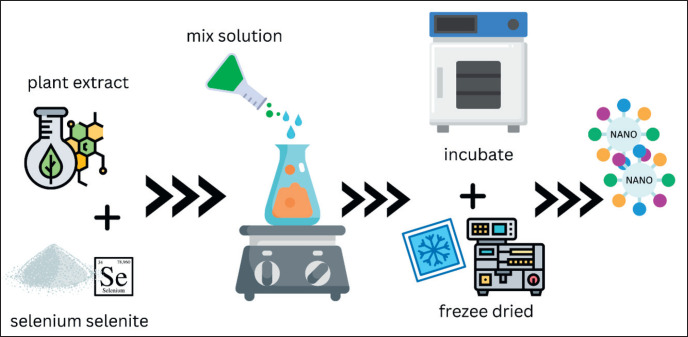
The green synthesis method produced nanomaterials (adapted from Jeevanandam [9]). The utilization of phenol in plants as a biocatalyst is the main principle in the preparation of nano-Se using the biosynthesis method. The phenol will break down and capture selenium at nano size and inhibit nano-Se from recombining by shrouding the nano selenium that has been formed.

Nano-Se can also be prepared using prokaryotic microorganisms, such as bacteria. Bacteria can be used as agents in nanomineral synthesis because they have several advantages, including easy maintenance, extracellular production, and short life cycles. The fabrication can use a group of Gram-negative bacteria of the genus Serratia isolated from the midgut of the insect *Stibara sp. *However, it can also use other bacteria, such as *Morganella morganii*, *Morganella psychotolerans*, *Pseudomonas stutzeri, Streptomyces sp., Escherichia coli, *and *P. stutzeri*. In addition, the production of nanominerals can also use fungi such as *Penicillium citrinum., Pseudomonas waksmanii, P. waksmanii, Fusarium oxysporum,* and the brown algae *Bifurcaria bifurcate* [58]. The green synthesis method produced nanomaterials presented in Figure 5.

Another new alternative method for biological methods is using plant extract as a biocatalyst on nanominerals, more commonly known as `green synthesis`. Utilizing plant extracts such as *Aloe barbedensis, Azadirachta indica,* and *Coriandrum sativum* [59], to produce nano-Se. Producing nano-Se using bio-catalyst such as *Bougainvillea spectabilis*, *Withania somnifera*, *Trigonella foenum-graecum,* and others [60–62]. The principle of this method is utilizing plant secondary metabolites and phytochemicals such as phenols and flavonoids to break minerals. It also prevents the agglomeration and deformation of nanominerals and allows the adsorption of phytochemicals on the surface of the nano substrate, which enhances the reaction rate of nanominerals [64,65].

Each nano-Se synthesis method has advantages and disadvantages, and its intended use determines its applicability. Physical methods typically necessitate expensive vacuum systems or apparatus to produce Nano. On the other hand, biological methods are more environmentally friendly due to the absence of corrosive compounds, but they require culture medium maintenance. In addition, chemical processes are preferred to produce nano-Se because they produce Nano of uniform size, are less expensive, and take less time [48].

### Nano-Se size distribution measurements (validation) and its morphological properties

Measurement using a particle size validation tool is required to confirm whether the particle size has reached nanosize. Testing of nano-Se can be done in various ways, including using a particle size analyzer, scanning electron microscope, transmission electron microscope (TEM), X-ray diffraction patterns, and others. The utilization of nanominerals could increase the bioavailability of each mineral by increasing cellular uptake and avoiding mineral antagonism. However, attention must be paid to its metabolism in the ruminant`s body to prevent potential toxicity [1]. Previous studies on nano-Se formulations as supplements [65] indicated that the nano-Se produced was in the form of a fine powder with a yield concentration of 68%; observations under a TEM were spherical in diameter between 30 and 80 nm; a zeta potential value of −22.8 mV; and a mineral concentration using X-ray dispersive energy analysis of 98.57 ± 0.48 as the final product.

Application of Nano-Se Supplementation on Ruminant Digestibility and Rumen Fermentation

The efficacy or efficiency of feed supplementation to maximize livestock productivity requires consideration of the effects of supplementation on rumen activity and digestibility rates. The absorption and utilization of nutrients from the feed ingested depend on the level of rumen fermentation, which is an exciting mechanism utilized by ruminants. Several previous studies have described the effect of nano-Se on rumen fermentation activity (Table 1). In general, nano-Se supplementation was found to elevate feed digestibility in the rumen, enhance the feed conversion rate, and increase total rumen volatile fatty acid (VFA) production [29,66,67].

Conventional forms of selenium supplements often have low absorption and potentially high toxicity. Then, nano-Se represents a method or processing of selenium, making it easier to absorb, but the release of active compounds is better controlled, especially in livestock with selenium deficiency, which can then be used as a booster agent to increase livestock productivity and reproduction [19].

In the rumen environment, many enzymatic structures and seleno-proteins, such as glutathione peroxidase-1, glutathione peroxidase-4, iodothyronine-5-deiodinase, and thioredoxin reductase-1, contained selenium as a significant component involved in antioxidant activity [14,68]. Glutathione S-transferase is known to be involved in enzyme activity and protects body tissues from oxidative damage [69]. This mechanism, Se supplementation, especially in the form of nano-Se, may also influence the microbial activity of ruminal fluid owing to its antioxidant properties and modulation of the rumen cell immune system [70]. The availability of nano-Se is known to increase rumen fermentation activity, feed digestibility [dry matter (DM), organic matter, and crude protein], and feed digestibility of processed extracts [29]. According to a previous study [71], nano-Se supplementation (20, 40, 60, and 80 mg nano-Se/kg DM) in wheat straw decreased the total amount of gas produced during fermentation along with increasing sodium selenite levels. By increasing the enzymatic activity of phenylalanine ammonium lyase, selenium alters the phenylpropanoid pathway in plant feed metabolism [72]. According to some studies, the addition of nano-Se increases the number of phenolic compounds in plant feed. The results of a study on the impact of nano-Se on VFA modification showed that, although it did not affect acetic acid formation, nano-Se addition increased the concentration of propionic acid and total VFA. Similar studies revealed that cattle diets contain a large amount of sodium selenite. Over time, they noticed a decrease in the acetate-to-propionate ratio [73,74].

The process of selenium absorption in the digestive tract of ruminants and non-ruminants is very different. Compared to non-ruminant animals, ruminant animals take longer to metabolize Se and tend to have lower Se absorption ability. Low selenium absorption in ruminants was believed to be caused by the reduction of dietary selenium to insoluble forms, such as elemental selenium or selenide in the rumen environment [20]. It is also because selenium is predigested by microbes in the rumen and reticulum before its sequestration in the abomasum and microbial digestion in the small intestine of ruminants so that selenium levels are absorbed in smaller amounts [74]. Thus, the administration of selenium in the form of nanominerals in ruminants is important [72].

High bioavailability on supplementation of nano-Se also affected the chemical character of nano-Se itself, especially production nano-Se using polymers having acidity characters (pH 4) that cannot be utilized in the rumen environment (pH 6) but are more easily broken down and utilized for absorption in the abomasum of the ruminant body (62% increase compared to the control) [72,75,76]. Other studies explain that increasing the bioavailability of nano-Se forms may make them safer and decrease the potential for toxicity, thereby reducing the potential for damage to livestock organs [77].

The availability of naturally absorbed nano-Se was altered by the presence of rumen microorganisms such as lactic acid bacteria, *Bifidobacteria*, and protozoa (ciliates) [78]. During the anaerobic respiration process, groups of bacteria and archaea use nano-Se as electron acceptors to produce nanospheres, which are insoluble forms of Se [71]. Another study stated that the addition of yeast-nano-Se also enhanced dominant bacterial phyla such as Bacteroidetes, Firmicutes, Fibrobacteres, Euryachaeota, Actinobacteria, Proteobacteria, Spirochaetes, and so on, resulting in increased VFA production [79]. Increased rumen microbia due to increased propionate rumen production, which enhanced the rate of utilization intake of Se by rumen microbiota; this mechanism also decreased methane production and acetate production, so the pH rumen did not decrease rapidly [81]. Overall addition of nano-Se tends to increase the activity and number of rumen microbes, which could then increase the production of total VFA, especially propionate, which will then cause a direct decrease in methane gas and cause an increase in ruminant energy reserves to improve livestock performance but reduce the potential for greenhouse gas production [74,79].

**Table 1.** Effect of nano-Se supplementation on rumen fermentation activity.

Treatment/element	Dosage/treatment	Livestock	Effect	Reference
Bionano-Se	25 and 50 ppm	Cattle (ongole cross)	The pH level was unaffected by the BioNano-Se treatment dosage. The overall gas values and a+b are not significantly affected by the dose of BioNano-Se.	[67]
Bionano-Se	25 and 50 ppm	Cattle (ongole cross)	The pH level was unaffected by the BioNano-Se treatment dosage. The overall gas values and a+b are not significantly affected by the dose of BioNano-Se.	[67]
Nano-Se	0, 1.5, 3, and 4.5 ppm	Cattle	The level of nano selenium additives does not affect the pH of the rumen fluid. The higher level of nano-selenium additives can increase the digestibility of dry matter *in vitro*. Total gas and CH4 production decreased with increasing nano-selenium levels. The addition of nano-Se did not affect total VFA or partial VFA.	[66]
Nano-Selenium	0, 0.1, 0.3, and 0.5 mg/Kg of DM	Dairy cow	The addition of Se linearly increased total VFA concentrations (*p* < 0.05) and the molar ratios of propionate and butyrate (*p* < 0.01), but also decreased rumen pH (*p* < 0.05), NH3-N concentration, and the acetate to propionate ratio (*p* < 0.01). In comparison to the treatment control group, the treatment 0.3 group had greater levels of propionate (*p* < 0.05). Acetate-to-propionate ratio and NH3-N concentration tended to be lower in the 0.3 group compared to the control group (*p* < 0.10). Se supplementation quadratically improved the digestibility of DM, OM, CP, NDF, and ADF and linearly increased the absorption of total Se (*p* < 0.05).	[80]
Nano-Se	4 gm/kg DM	Sheep	Increased propionate acid, total volatile fatty acids, reduced ammonia nitrogen, improved dry matter, and neutral detergent fiber digestibility	[29]
Nano-Se	0, 0.3, 3, and 6 gm/sheep/day	Sheep	• As compared to the control and 6 g of Se treatments, the digestibility of DM, organic matter (OM), crude protein (CP), ether extract (EE), NDF, and ADF was higher for the 0.3 and 3 of Se treatments. • With increasing nano-Se supplementation, mean ruminal pH and ammonia N concentration quadratically decreased. Acetate and butyrate‘s molar proportions were unaffected, but propionate‘s grew linearly with increasing nano-Se supplementation. The ratio of acetate to propionate therefore decreased linearly and quadratically. The amount of total ruminal VFA rose quadratically and linearly with increasing nano-Se supplementation.	[28]
Nano-Se yeast	2.4–4.8 mg/kg DM feed	Guizho goat	Increased microbe population Increased VFA yield	[79]

High bioavailability on nano-Se is possible because digestive mucosa is a possible passageway for nano-Se, which might penetrate via either paracellularly (i.e., neighboring cells) or transcellularly (between cells) pathways. The firm connections (pore diameter between 0.3 and 1.0 mm) among tissue cells and the small region between cells (paracellular at physiological conditions) inhibit the first route. Transcytosis, the process by which nano-Se are transported across cell membranes, begins with endocytosis at the cell’s apical membrane. Once within the cell, nano-Se are taken to the basolateral pole for release [46,76], where they can do their damage. In contrast to the natural form, which is poorly absorbed by cells due to its huge particle size, Nano are rapidly absorbed because they can penetrate through the stomach wall and diffuse into the body’s cells at a much faster rate. With a far higher surface area to volume ratio and more extensive contact with mucosal tissues and cells than the original form. The amount of time that nanominerals spend in the Gastro intestine tract is proportional to how well they are absorbed into the mucosal surface [1].

### Application of Nano-Se Supplementation to Ruminant Productivity

For the use of nanominerals in ruminants, it is necessary to consider the results of the structural form of the nanomineral, to improve feed and food-derived quality from animals, or to otherwise enhance ruminants’ production and health [13]. Trace mineral nanomaterials have already shown impressive results when used as animal feed supplements in ruminants such as selenium [1,14,76,82]. A brief review was conducted with the literature study presented in Table 2.

Nano-Se has functional characteristics and is essential in ruminant livestock production systems, especially in the feed digestibility system [29]. The impact of nano-Se supplementation in the digestive tract, as antioxidant and antibacterial [56], can enhance the immune system in the livestock body [83], which in turn could affect productivity both in terms of growth and reproduction [84]. In ruminant production, the functions of nano-Se are increasing body weight [85], increasing livestock productivity such as increasing yield weight or milk yield [14,28–30], and increasing feed intake and TDN [31,86]. Also, nano-Se supplementation in pregnant sows was able to increase superoxide dismutase, catalase, and superoxide dismutase in serum and liver while also increasing the activity of Immunoglobulin A and Immunoglobulin B in serum and liver to increase antioxidants and immunity due to intrauterine growth retardation causing death in newborn piglets or reduced piglet mortality [87], which is expected to have the same function in ruminants.

Based on the previous research, it was demonstrated that there was a difference in basal feed consumption in the presence of nano-Se supplementation, increasing feed consumption in non- and supplemented nano-Se by 200 gm/day in goats [28]. However, different types of nano-Se supplementation did not influence the consumption in these groups of goats. In the grower phase (90–180 days of age), where goats are in weaning condition, they are highly dependent on feed consumption to optimize their productivity, especially on growth rate. The higher the feed consumption, the better and faster the potential growth rate. This is evident from the final weaning weight (180 days), which is also in line with the level of feed consumption in the treatment group. Another study determined that there was a significant difference (*p* < 0.05) in the control group with the nano-Se supplementation group by 3–4 kg. However, different nano-Se consumption did not affect the final weight in the goat group, the difference in nano-Se supplementation was not significantly different in final weight. Thus, in terms of average daily gain (ADG), there was also a significant difference between the control group and the Se supplementation group (*p* < 0.05) by 25–35 gm/day, and Se-Yeast and Se-Nano supplementation resulted in higher ADG (*p* < 0.05) than regular Se supplementation SS, by 10 gm/day.

**Table 2.** Effect of nano-selenium supplementation on ruminant productivity.

Element	Dosage/treatment	Livestock	Effects	References
Nano-Se	Taihang black ram	Addition 0.32 mg/kg DM nano-Se Addition Se-Yeast 0.03 mg/kg DM Addition nano-Se 0.034 mg/kg DM	There is a significant increase in blood and serum Se content per 90 days Significant increase in ADG Control 49,90 gm/hari Se 75,34 gm/hari Se-yeast 85,66 gm/hari Nano-Se 84,67 gm/hari	[28]
Nano-Se-Yeast	Italian Apennine ram	0.03–0.045 mg/kg DM	Enhanced ADG 163.6–173.4 gm/day Increased Se contain in meat (L. dorsi) 0.66%–0.84%	[88]
Nano-Se-Yeast	Corriedale ram	0.035–0.35 ppm	Enhanced final body weight 26.6%	[89]
Nano-Se	Taihanng black goat	0.1–1 mg/kg DM feed basa;	Enhanced ADG 86–94.5g/day	[90]

The results of research by Shi et al [28] showed that Se-nano is very effective and has great potential in efforts to increase goat productivity in the grower phase, namely accelerating the growth rate. Nano-Se supplementation with basic or yeast in different levels did not have a significant effect on the production parameters of male and female Italian Apennine sheep, including ADG, slaughter weight, carcass weight, carcass percentage, and meat quality. However, the results of the selenium content test in meat showed a real and significant difference (*p* < 0.05), in the control group with nano-Se supplementation of 10%–50%, and also between the forms of Se supplementation significantly different (*p* < 0.05) by 20%. Regular nano-Se supplementation compared to Se yeast significantly reduced (*p* < 0.05) the concentration of fatty acids (ΣFAs) in meat fat (Longissimus muscle) from 14.3 to 9.3 mg/g [89]. Thus, Se-yeast supplementation can improve lamb meat quality by increasing its nutritional value. In addition to being caused by the ability as an antioxidant, the increase in ruminant productivity in the addition of nano-Se is also facilitated by the function of selenium in insulin growth factor-1 metabolism, which is very influential in cell proliferation and growth factor ability and plays a role in insulin metabolism in the liver [91].

A recommended dietary intake of selenium for cattle has been estimated at 100 μg/kg DM for beef cattle and 300 μg/kg DM for dairy cattle. For calves, the daily selenium requirement is set at 100 μg/kg DM [92]. In contrast, the addition of selenium, both organic and inorganic, at a concentration of 0.3 mg/kg in feed mixtures for sheep and lambs has been shown to have a positive impact on the metabolism and immune function of lambs without adverse effects [93]. In addition, a selenium level of 0.4 mg/kg DM is considered suitable for the early growth and development of Tibetan lambs [94].

### Application of Nano-Se Supplementation to Ruminant Reproduction

Selenium (Se) is an essential trace mineral that plays an important role in reproduction and overall health, is necessary for growth and fertility in animals, and is also for the prevention of a variety of disease conditions [95]. Many studies have shown that mineral nutrition has an important role in ruminants’ reproductive performance, and the relationship between nutrition and physiology has played a key role in recent years [96]. Trace element deficiencies in the diet have been reported to alter various aspects of ruminants’ reproductive physiology [97]. In recent years, the use of Nano has begun to dominate all scientific fields, including livestock reproduction, and facilitates specialized improvements in this regard while offering many innovative interventions, including the use of feed minerals in the improvement of livestock reproductive performance [1]. Several previous studies have described the effect of nano-Se supplementation on ruminant reproduction (Table 3).

Se is involved in several important biological processes, including reproduction, circulation, hormone production, and the generation of thyroxine and thyroid hormone. Se also passes through the placenta and milk from mother to fetus; therefore, the Se status of a female immediately influences the health and survival of animal offspring [14,100–102]. According to previous research, a marginal lack of selenium can result in reduced fertility, silent heat, cystic ovaries, and the birth of sick/unhealthy offspring (kids/lambs/calf) with inadequate immunity as a result of the enhancement of immunoglobin G (IgG) pathways [103]. Studies on reproductive performance indicated that injecting ewes with selenium for a total of 8 weeks (4 weeks before parturition and then again during the final 4 weeks of gestation) resulted in a 32% increase in the percentage of lambs born when compared to the percentage of lambs born to controls [104]. When ewes were given one selenium injection before the time of mating, a previous study reported a rise in the number of ewes that were in heat, as well as increased pregnancy rates, lambing rates, and twin rates. This was the case even though the ewes had not been exposed to any further selenium [105].

**Table 3.** Effect of nano selenium supplementation on ruminant reproduction.

Element	Dosage/treatment	Livestock	Effects	References
Nano-Se	0,1–0,2 mg/kg oral	Romanov crossbred ewes	Pregnant survival litter size 100% in comparison with control group.	[98]
Nano-Se	0.6 mg/kg/day	Khalkhali goats	There was a significant difference between the groups in serum IgG concentration, colostrum IgG, and blood IgG concentration of kids (*p* < 0.05)	[14]
Nano-Se	0.3 mg/kg DM intake	Goats	Increased Se in testes Increased GPx and ATPase activities Lower abnormality ejaculate	[28]
Nano-Se	0.2 mg/kg oral	Ossimi ewes	had higher (*p* < 0.01) period in estrus (estrus duration) and it took a shorter (*p* < 0.01) period to come into estrus than control group in the first service and post-partum after the intra vaginal sponges withdrawn. the estrus signs had more (*p* < 0.05) intense in the ewes treated with organic selenium (69%) and nano- selenium (57%) than control group (41%). While	[99]

Nano-Se has begun to be explored in ruminants due to its high bioavailability, high catalytic effectiveness, high absorption capacity, and low toxicity. This is in comparison to the basic form of selenium, which has a low level of bioavailability. Nano-Se have been utilized in many different investigations as anti-reactive oxygen species (ROS) agents to protect against oxidative damage [34]. According to previous research, the balance of ROS and antioxidants in female mammalian species is known to affect endometrial changes in different luteal phases, folliculogenesis, ovulation, fertilization, placenta growth, embryogenesis, and implantation. This mechanism is expected to affect endometrial changes in different luteal phases. In its activity as an antioxidant precursor, nano-Se will preclude damage from free radicals by protecting the cell nucleus (deoxyribonucleic acid) from oxidative reactions, lowering the concentration of thiobarbituric acid reactive substances (an indicator of oxidative stress), reducing the potential toxicity of oxidative NADPH enzyme activation, and increasing GPx production in the cell [91]. Some studies retriever that supplementation of nano-Se significantly increased both follicles and corpus luteum size and number which was caused by the follicle stimulating hormone stimulate mechanism after the addition of nano-Se which is representative of the higher reproductive hormones rather than the control. Estrus responses and fertility rate then kidding rate also increased by ewes feeding nano-Se; this is likely to occur by antioxidant activity from nano-Se, which stimulated GPx to detoxify extracellular radicals.

In another study, male ruminants fed nano-Se 0.3 mg/kg DM for 12 weeks from the time of weaning to the time of puberty exhibited a significantly elevated selenium level in the testes, as well as GPx and adenosine triphosphate (ATP) ase activities in the ejaculate when compared with the control group. This was true even though the amount of nano-Se consumed was the same. The addition of selenium did not change the ejaculate quality (volume, density, motility, or pH), but the percentage of aberrant spermatozoa in the control goat group was much higher than that in the nano-Se group. This difference was statistically significant. TEM was used to discover that selenium-deficient goats had damaged sperm plasma membranes, as well as anomalies in the mitochondrial centers of their spermatozoa. Another finding stated administration of nano-Se on ram or buck feed could increase semen motility and intact membrane cells and reduce declined acromose integrity compared with control. This phenomenon is due to antioxidant activity in nano-Se such as the GPx enzyme, which counters well ROS production in sperm cells [106].

### Conclusion

In conclusion, the utility of using Nano in ruminant feed production shows a good trend and development, especially the utilization as a feed supplementation in addition to maximizing the bioavailability of these trace essential minerals. Fabrication and application of nanominerals in the form of Se as an effort to increase the productivity of ruminants have been widely carried out; the newest and safest was by using a biological method that produces nanominerals by employing plant extract as a biocatalyzer and prevents any reunification both in nano-Se. The addition of nano-Se in a certain level not only still maintains the rumen environment but also could create excellent rumen conditions, which then resulted in increasing VFA production and elevated ruminant production in general. Also, the addition of nano-Se increased feed intake and digestibility of both DM, organic matter, crude protein, and total digestible nutrients to increase energy reserves for livestock growth. It is proven that nano-Se supplementation in feed can increase body weight and milk yield in ruminants. In reproduction fields, nano-Se plays as an antioxidant agent, which is recently known to improve various reproductive parameters so that it can also improve reproductive efficiency in ruminants. The results of this review imply that there are wide open opportunities for the potential utilization of Nano in the process of supplementing Se in ruminant feed more comprehensively with various combinations of fabrication methods, supplementation methods, and supplementation levels that are tested *in vivo*.

## List of abbreviations

ADG, Average daily gain; ATP, Adenosine triphosphate; CAT, Catalase; DM, Dry matter; gm/day, Gram per day; Kg, Kilogram; mg, Milligram; mm, Millimeter; mol/l, Molarity per liter; Nano, Nanoparticle/nanotechnology; ROS, Reactive oxygen species; Se, Selenium; TDN, Total digestible nutrient; TEM, Transmission electron microscope; VFA, Volatile fatty acid.

## References

[1] Abdelnour SA, Alagawany M, Hashem NM, Farag MR, Alghamdi ES, Hassan FU (2021). Nanominerals: fabrication methods, benefits and hazards, and their applications in ruminants with special reference to selenium and zinc nanoparticles. Animals.

[2] El Hack MEA, Alagawany M, Farag MR, Arif M, Emam M, Dhama K (2017). Nutritional and pharmaceutical applications of nanotechnology: trends and advances. Int J Pharmacol.

[3] Hashem NM, Sallam SM (2020). Reproductive performance of goats treated with free gonadorelin or nanoconjugated gonadorelin at estrus. Domest Anim Endocrinol.

[4] Osama E, El Sheikh SMA, Khairy MH, Galal AAA (2020). Nanoparticles and their potential applications in veterinary medicine. J Adv Vet Res.

[5] Reddy PRK, Yasaswini D, Reddy PPR, Zeineldin M, Adegbeye MJ, Hyder I (2020). Applications, challenges, and strategies in the use of nanoparticles as feed additives in equine nutrition. Vet World.

[6] Khan Y, Sadia H, Ali Shah SZ, Khan MN, Shah AA, Ullah N (2022). Classification, synthetic, and characterization approaches to nanoparticles, and their applications in various fields of nanotechnology: a review. Catalysts.

[7] Ingale SV, Wagh PB, Bandyopadhyay D, Singh IK, Tewari R, Gupta SC Synthesis of nanosized platinum based catalyst using sol-gel process. IOP Conf Ser Mater Sci Eng 2022.

[8] Lestari SR, Prastita N, Maslikah SI, Sunaryono Gofur A, Fajaroh F (2023). Development of self nanoemulsifying drug delivery system (SNEDDS) to improve antioxidant activity of single garlic extract (*Allium sativum* L.). AIP Conf Proc.

[9] Jeevanandam J, Chan YS, Danquah MK (2019). Zebrafish as a model organism to study nanomaterial toxicity. Emerg Sci J.

[10] Baig MMFA, Zhang C, Akhtar MF, Saleem A, Mudassir J (2021). The effective transfection of a low dose of negatively charged drug-loaded DNA-nanocarriers into cancer cells via scavenger receptors. J Pharm Analys.

[11] Bhagat S, Singh S (2022). Nanominerals in nutrition: recent developments, present burning issues and future perspectives. Food Res Int.

[12] Gopi M, Pearlin B, Kumar RD, Shanmathy M, Prabakar G (2017). Role of nanoparticles in animal and poultry nutrition: modes of action and applications in formulating feed additives and food processing. Int J Pharmacol.

[13] Idamokoro EM, Hosu YS (2022). Global research trends on the use of nanotechnology to boost meat production: a scientometric analysis. Front Res Metrics Analyt.

[14] Kachuee R, Abdi Benemar H, Mansoori Y, Seifdavati J, Elghandour MMMY (2019). Effects of sodium selenite, L-selenomethionine, and selenium nanoparticles during late pregnancy on selenium, zinc, copper, and iron concentrations in Khalkhali goats and their kids. Biol Trace Element Res.

[15] Malyugina S, Skalickova S, Skladanka J, Slama P, Horky P (2021). Biogenic selenium nanoparticles in animal nutrition: a review. Agriculture (Switzerland).

[16] Herdt TH, Hoff B (2011). The use of blood analysis to evaluate trace mineral status in ruminant livestock. Vet Clin North Am Food Anim Pract.

[17] Kendall NR, MacKenzie AM, Telfer SB (2012). The trace element and humoral immune response of lambs administered a zinc, cobalt and selenium soluble glass bolus. Livest Sci.

[18] Spears JW, Weiss WP (2014). Mineral and vitamin nutrition in ruminants 1. Professional Anim Sci.

[19] Hosnedlova B, Kepinska M, Skalickova S, Fernandez C, Ruttkay Nedecky B, Donald Malevu T (2017). A summary of new findings on the biological effects of selenium in selected animal species—a critical review. Int J Mol Sci.

[20] Badgar K, Prokisch J (2020). The effects of selenium nanoparticles (SeNPs) on ruminant. Proc Mongolian Acad Sci.

[21] Stocco G, Summer A, Malacarne M, Cecchinato A, Bittante G (2019). Detailed macro- and micromineral profile of milk: effects of herd productivity, parity, and stage of lactation of cows of 6 dairy and dual-purpose breeds. J Dairy Sci.

[22] Xie J, Shen Z, Anraku Y, Kataoka K, Chen X (2019). Nanomaterial-based blood-brain-barrier (BBB) crossing strategies. Biomaterials.

[23] Tan Q, Wu C, Li L, Shao W, Luo M (2022). Nanomaterial-based prosthetic limbs for disability mobility assistance: a review of recent advances. J Nanomat.

[24] Ramachandraiah K, Choi MJ, Hong GP (2018). Micro- and nano-scaled materials for strategy-based applications in innovative livestock products: a review. Trends Food Sci Technol.

[25] Sevim B, Cufadar Y (2021). Effects of essential oils and their combinations added to broiler diets on the mineral contents of some tissues and bone breaking strength. *Rocz Nauk Pol Tow Zootech*.

[26] Khajeh Bami M, Afsharmanesh M, Espahbodi M (2022). Dietary supplementation with biosynthesised nano-selenium affects growth, carcass characteristics, meat quality and blood parameters of broiler chickens. Anim Prod Sci.

[27] Dawood MAO, El Basuini MF, Yilmaz S, Abdel-Latif HMR, Kari ZA, Abdul Razab MKA (2021). Selenium nanoparticles as a natural antioxidant and metabolic regulator in aquaculture: a review. Antioxidants.

[28] Shi L, Xun W, Yue W, Zhang C, Ren Y, Liu Q (2011). Effect of elemental nano-selenium on feed digestibility, rumen fermentation, and purine derivatives in sheep. Anim Feed Sci Technol.

[29] Xun W, Shi L, Yue W, Zhang C, Ren Y, Liu Q (2012). Effect of high-dose nano-selenium and selenium-yeast on feed digestibility, rumen fermentation, and purine derivatives in sheep. Biol Trace Element Res.

[30] Tsiplakou E, Mitsiopoulou C, Karaiskou C, Simoni M, Pappas AC, Righi F (2021). Sesame meal, vitamin E and selenium influence goats’ antioxidant status. Antioxidants.

[31] Ibrahim M, Ibrahim E, Mohamed M (2018). Productive performance of ossimi ewes post-lambing as affected by selenium yeast and/or vitamin e supplemented rations under two different housing types. J Anim Poult Prod.

[32] Siswoyo P, Tafsin M, Handarini R (2018). Potential reproduction and response of selenium and zinc mineral supplementation on quality of goat Samosir semen. IOP Conf Ser Earth Environ Sci.

[33] Ghafarizadeh AA, Vaezi G, Shariatzadeh MA, Malekirad AA (2018). Effect of *in vitro* selenium supplementation on sperm quality in asthenoteratozoospermic men. Andrologia.

[34] Khalil HS, Mansour AT, Goda AMA, Omar EA (2019). Effect of selenium yeast supplementation on growth performance, feed utilization, lipid profile, liver and intestine histological changes, and economic benefit in meagre, Argyrosomus regius, fingerlings. Aquaculture.

[35] Nateq S, Moghaddam G, Alijani S, Behnam M (2020). The effects of different levels of Nano selenium on the quality of frozen-thawed sperm in ram. J Appl Anim Res.

[36] Talebi E, Ghazanfarpour H, Ghazanfarpoor R, Bouchentouf S, Khosravinezhad M (2021). Application of selenium nanoparticles on sperm quantity indicators in Wistar rat. Nephro-Urol Monthly.

[37] Han D, Xiong S, Jia W, Chen S, Wei Y, Shao H (2021). Separation of selenium species in plant tissues by high performance liquid chromatography-ultraviolet treatment-hydride generation atomic fluorescence spectrometry using various mobile phases. Biotechnol Biotechnol Equip.

[38] Dahlen CR, Reynolds LP, Caton JS (2022). Selenium supplementation and pregnancy outcomes. Front Nutr.

[39] Boostani A, Sadeghi AA, Mousavi SN, Chamani M, Kashan N (2015). Effects of organic, inorganic, and nano-Se on growth performance, antioxidant capacity, cellular and humoral immune responses in broiler chickens exposed to oxidative stress. Livest Sci.

[40] Jamal Rajab W, Rudiansyah M, Kadhim MM, Tolmasovich Shamsiev A, Prakaash AS, Hadi Lafta M (2023). The role of selenium on the status of mineral elements and some blood parameters of blood serum of lambs. Arch Razi Instit.

[41] Khabatova VV, Serov DA, Tikhonova IV, Astashev ME, Nagaev EI, Sarimov RM (2022). Selenium nanoparticles can influence the immune response due to interactions with antibodies and modulation of the physiological state of granulocytes. Pharmaceutics.

[42] Higuchi A, Inoue H, Kaneko Y, Oonishi E, Tsubota K (2016). Selenium-binding lactoferrin is taken into corneal epithelial cells by a receptor and prevents corneal damage in dry eye model animals. Sci Rep.

[43] Shabani R, Fakhraei J, Yarahmadi HM, Seidavi A (2019). Effect of different sources of selenium on performance and characteristics of immune system of broiler chickens. Rev Brasil Zoot.

[44] Ren Z, Wu Q, Deng H, Yu Y, Tang W, Deng Y (2021). Effects of selenium on the immunotoxicity of subacute arsenic poisoning in chickens. Biol Trace Elem Res.

[45] Alagawany M, Qattan SYA, Attia YA, El-Saadony MT, Elnesr SS, Mahmoud MA (2021). Use of chemical nano-selenium as an antibacterial and antifungal agent in quail diets and its effect on growth, carcasses, antioxidant, immunity and caecal microbes. Animals.

[46] Ferro C, Florindo HF, Santos HA (2021). Selenium nanoparticles for biomedical applications: from development and characterization to therapeutics. Adv Healthc Mat.

[47] Nandihalli N, Gregory DH, Mori T (2022). Energy-saving pathways for thermoelectric nanomaterial synthesis: hydrothermal/solvothermal, microwave-assisted, solution-based, and powder processing. Adv Sci.

[48] Sarathi Swain P, Prusty S, Bala Nageswara Rao S, Rajendran D, Kumar Patra A, Patra AK (2021). Essential nanominerals and other nanomaterials in poultry nutrition and production. Advances in Poultry Nutrition Research.

[49] Raje K, Ojha S, Rawat C, Mishra A, Munde VK, Chaudhary SK (2018). Impact of supplementation of mineral nano particles on growth performance and health status of animals: a review. J Entomol Zool Stud.

[50] Hidayat C (2022). Efektivitas penggunaan nanomineral pada pakan terhadap peningkatan performa ayam: review. Indones J Anim Sci.

[51] Nyabadza A, Vazquez M, Brabazon D (2023). A review of bimetallic and monometallic nanoparticle synthesis via laser ablation in liquid. Crystals.

[52] Rigopoulos I, Kyriakou L, Vasiliades MA, Kyratsi T, Efstathiou AM, Ioannou I (2021). Improving the carbonation of air lime mortars at ambient conditions via the incorporation of ball-milled quarry waste. Construct Build Mat.

[53] Liao X, Xie H, Liao B, Hou S, Yu Y, Fan X (2022). Ball milling induced strong polarization electric fields in Cu3B2O6 crystals for high efficiency piezocatalysis. Nano Energy.

[54] Rane AV, Kanny K, Abitha VK, Thomas S, Thomas S (2018). Methods for synthesis of nanoparticles and fabrication of nanocomposites. Synthesis Inorganic. Nanomat Adv Key Technol.

[55] Sharif A, Farid N (2022). Ultrashort laser sintering of metal nanoparticles: a review. Results Eng.

[56] Amendola V, Meneghetti M (2009). Laser ablation synthesis in solution and size manipulation of noble metal nanoparticles. Phys Chem Chem Phys.

[57] Apryatina KV, Murach EI, Amarantov SV, Erlykina EI, Veselov VS, Smirnova LA (2022). Synthesis of a bioactive composition of chitosan–selenium nanoparticles. Appl Biochem Microbiol.

[58] Shoeibi S, Mashreghi M (2017). Biosynthesis of selenium nanoparticles using *Enterococcus faecalis* and evaluation of their antibacterial activities. J Trace Elements Med Biol.

[59] Abbas HS, Krishnan A, Kotakonda M (2020). Fabrication of iron oxide/zinc oxide nanocomposite using creeper blepharis maderaspatensis extract and their antimicrobial activity. Front Bioeng Biotechnol.

[60] Pyrzynska K, Sentkowska A (2022). Biosynthesis of selenium nanoparticles using plant extracts. J Nanostruct Chem.

[61] Letchumanan D, Sok SPM, Ibrahim S, Nagoor NH, Arshad NM (2021). Plant-based biosynthesis of copper/copper oxide nanoparticles: an update on their applications in biomedicine, mechanisms, and toxicity. Biomolecules.

[62] Singh A, Singh NB, Hussain I, Singh H, Yadav V, Singh SC (2016). Green synthesis of nano zinc oxide and evaluation of its impact on germination and metabolic activity of Solanum lycopersicum. J Biotechnol.

[63] Aguilar MS, Esparza R, Rosas G (2019). Synthesis of Cu nanoparticles by chemical reduction method. Transact Nonferr Metals Soc China.

[64] Nasrollahzadeh M, Sajadi SM, Issaabadi Z, Sajjadi M (2019). Biological sources used in green nanotechnology. Interface Sci Technol.

[65] Arulnathan N, Karunakaran R, Balakrishanan V, Chellapandian M, Geetha K, Sabareeswaran A (2017). *In vitro* cytotoxicity assessment of nano selenium in mice for its biocompatibility as feed supplement. Indian Vet J.

[66] Campos Montiel RG (2022). Decrease of greenhouse gases during an *in vitro* ruminal digestibility test of forage (*Festuca arundinacea*) conditioned with selenium nanoparticles. Nanomaterials.

[67] Nurfitriani RA, Jayanegara A, Kumalasari NR, Ratnakomala S, Rohmatussolihat Sari NF (2023). Effects of bionanomineral selenium (bionano-se) and probiotics inclusion to ration on *in vitro* rumen fermentation characteristics. J Anim Plant Sci.

[68] Lee MRF, Fleming HR, Whittington F, Hodgson C, Suraj PT, Davies DR (2019). The potential of silage lactic acid bacteria-derived nano-selenium as a dietary supplement in sheep. Anim Prod Sci.

[69] Neve J (2002). Selenium as a “nutraceutical”: how to conciliate physiological and supra-nutritional effects for an essential trace element. Curr Opin Clin Nutr Metab Care.

[70] Chauhan SS, Ponnampalam EN, Celi P, Hopkins DL, Leury BJ, Dunshea FR (2016). High dietary vitamin E and selenium improves feed intake and weight gain of finisher lambs and maintains redox homeostasis under hot conditions. Small Rum Res.

[71] Campos Montiel R (2018). Inhibitory effect of selenium concentrations on microbial activity during oat hay *in vitro* rumen fermentation. Agrociencia.

[72] Mendoza AB (2020). Ionic selenium and nanoselenium as biofortifiers and stimulators of plant metabolism. Agronomy.

[73] Zhang ZD, Wang C, Du HS, Liu Q, Guo G, Huo WJ (2020). Effects of sodium selenite and coated sodium selenite on lactation performance, total tract nutrient digestion and rumen fermentation in Holstein dairy cows. Animal.

[74] Cui X, Wang Z, Tan Y, Chang S, Zheng H, Wang H (2021). Selenium yeast dietary supplement affects rumen bacterial population dynamics and fermentation parameters of tibetan sheep (*Ovis aries*) in alpine meadow. Front Microbiol.

[75] Satarzadeh N, Sadeghi Dousari A, Amirheidari B, Shakibaie M, Ramezani Sarbandi A, Forootanfar H (2023). An insight into biofabrication of selenium nanostructures and their biomedical application. 3 Biotech.

[76] Hosnedlova B, Kepinska M, Skalickova S, Fernandez C, Ruttkay Nedecky B, Peng Q (2018). Nano-selenium and its nanomedicine applications: a critical review. Int J Nanomed.

[77] Wang MQ, Wang C, Li H, Du YJ, Tao WJ, Ye SS (2012). Effects of chromium-loaded chitosan nanoparticles on growth, blood metabolites, immune traits and tissue chromium in finishing pigs. Biol Trace Elem Res.

[78] Galbraith ML, Vorachek WR, Estill CT, Whanger PD, Bobe G, Davis TZ (2016). Rumen microorganisms decrease bioavailability of inorganic selenium supplements. Biol Trace Elem Res.

[79] Tian J, Zhang X, Zhou Z, Liu H, Fu Y, Zhang J (2018). Microstructure and thermoelectric properties of n type nanocomposite. J Funct Mat.

[80] Wei JY, Wang J, Liu W, Zhang KZ, Sun P (2019). Effects of different selenium supplements on rumen fermentation and apparent nutrient and selenium digestibility of mid-lactation dairy cows. J Dairy Sci.

[81] Hendawy AO, Sugimura S, Sato K, Mansour MM, Abd El Aziz AH, Samir H (2022). Effects of selenium supplementation on rumen microbiota, rumen fermentation and apparent nutrient digestibility of ruminant animals: a review. Fermentation.

[82] Mohammadi A, Ghazanfari S, Sharifi SD (2019). Comparative effects of dietary organic, inorganic, and nano-selenium complexes and rosemary essential oil on performance, meat quality and selenium deposition in muscles of broiler chickens. Livest Sci.

[83] Kojouri GA, Sadeghian S, Mohebbi A, Dezfouli MRM (2012). The effects of oral consumption of selenium nanoparticles on chemotactic and respiratory burst activities of neutrophils in comparison with sodium selenite in sheep. Biol Trace Elem Res.

[84] Wilde D (2006). Influence of macro and micro minerals in the peri-parturient period on fertility in dairy cattle. Anim Reprod Sci.

[85] Hisham Khalifa H, Safwat M, El Sysy M, Al-Metwaly M (2016). Effect of selenium and vitamin E supplementation as a nutritional treatment for some physiological and productive traits of Holstein dairy cows under Egyptian summer conditions. J Egypt Acad Soc Environ Dev D Environ Stud.

[86] Ibrahim E, Mohamed M (2018). Effect of different dietary selenium sources supplementation on nutrient digestibility, productive performance and some serum biochemical indices in sheep. Egypt J Nutr Feeds.

[87] Liu S, Yu H, Li P, Wang C, Liu G, Zhang X (2022). Dietary nano-selenium alleviated intestinal damage of juvenile grass carp (*Ctenopharyngodon idella*) induced by high-fat diet: insight from intestinal morphology, tight junction, inflammation, anti-oxidization and intestinal microbiota. Anim Nutr.

[88] Vignola G, Lambertini L, Mazzone G, Giammarco M, Tassinari M, Martelli G (2009). Effects of selenium source and level of supplementation on the performance and meat quality of lambs. Meat Sci.

[89] Jaworska D, Czauderna M, Przybylski W, Rozbicka Wieczorek AJ (2016). Sensory quality and chemical composition of meat from lambs fed diets enriched with fish and rapeseed oils, carnosic acid and seleno-compounds. Meat Sci.

[90] Yue W, Zhang C, Shi L, Ren Y, Jiang Y, Kleemann DO (2009). Effect of supplemental selenomethionine on growth performance and serum antioxidant status in Taihang black goats. Asian-Australas J Anim Sci.

[91] Çiçek S, Özoğul F (2021). Effects of selenium nanoparticles on growth performance, hematological, serum biochemical parameters, and antioxidant status in fish. Anim Feed Sci Technol.

[92] Mehdi Y, Dufrasne I (2016). Selenium in cattle: a review. Molecules.

[93] Novoselec J, Novoselec M (2022). The effect of maternal dietary selenium supplementation on blood antioxidant and metabolic status of ewes and their lambs. Antioxidants.

[94] Wang B, Zhang C, Wang J, An H, Guo Z, Lv Z (2019). Study of nano–Cu/SiO 2 catalysts for highly selective hydrogenation of acetophenone. Appl Organomet Chem.

[95] Ziaei N (2015). Effect of selenium and vitamin E supplementation on reproductive indices and biochemical metabolites in Raieni goats. J Appl Anim Res.

[96] Argüello A (2011). Trends in goat research, a review. J Appl Anim Res.

[97] Rojo R, Salem AZM, Tinoco JL (2011). Trace elements in sheep and goats reproduction: a review. Trop Subtrop Agroecosyst.

[98] Salam A, EL-Shamaa I, Metwally A, El Hewaty A, Mahmoud T, Zommara M (2021). Effect of selenium adminstration on reproductive outcome and biochemical parameters to ewes and their lambs. J Anim Poult Prod.

[99] Abd-Elkareim M, Ali M, Fahmy S, Hussein A (2021). Reproductive performance and lamb’s birth weight in Ossimi ewes treated with organic selenium and nano-selenium under Upper Egyptian condition. Arch Agric Sci J.

[100] Yatoo MI, Saxena A, Kumar P, Shekhar P, Dimri U, Sharma MC (2013). Evaluation of serum mineral status of sheep and its relation to hormones. Vet Pract.

[101] Yatoo MI, Saxena A, Kumar P, Gugjoo MB, Dimri U, Sharma MC (2013). Evaluation of serum mineral status and hormone profile in goats and some of their inter-relations. Vet World.

[102] Khalil HM, Abdallah AG, El-Sahn AA, El-Saadany AS, Nehad A, Abd El-Salam A (2014). Effect of supplementing organic trace minerals (zinc, manganese, iron, copper and selenium) on semen quality, reproductive performance and immune response of Gimmizah cocks. Egypt Poult Sci J.

[103] Neal ES, Hofstee P, Askew MR, Kent NL, Bartho LA, Perkins AV (2021). Maternal selenium deficiency in mice promotes sex-specific changes to urine flow and renal expression of mitochondrial proteins in adult offspring. Physiol Rep.

[104] Gabryszuk M, Klewiec J (2002). Effect of injecting 2- and 3-year-old ewes with selenium and selenium-vitamin E on reproduction and rearing of lambs. Small Rum Res.

[105] Koyuncu M, Yerlikaya H (2007). Effect of selenium-vitamin E injections of ewes on reproduction and growth of their lambs. South Afr J Anim Sci.

[106] Shahin MA, Khalil WA, Saadeldin IM, Swelum AAA, El-Harairy MA (2020). Comparison between the effects of adding vitamins, trace elements, and nanoparticles to SHOTOR extender on the cryopreservation of dromedary camel epididymal spermatozoa. Animals.

